# Nanocellulose separation from barley straw via ultrasound-assisted choline chloride – Formic acid deep eutectic solvent pretreatment and high-intensity ultrasonication

**DOI:** 10.1016/j.ultsonch.2024.107048

**Published:** 2024-08-30

**Authors:** Dileswar Pradhan, Swarna Jaiswal, Brijesh K. Tiwari, Amit K. Jaiswal

**Affiliations:** a Sustainable Packaging and Bioproducts Research (SPBR), School of Food Science and Environmental Health, Faculty of Sciences and Health, Technological University Dublin—City Campus, Central Quad, Grangegorman, Dublin, Ireland; b Sustainability and Health Research Hub (SHRH), Technological University Dublin—City Campus, Grangegorman, Dublin, Ireland; c Teagasc Food Research Centre, Ashtown, Dublin, Ireland

**Keywords:** Agricultural waste, Deep eutectic solvent, Ultrasound, Lignin, Nanocellulose

## Abstract

The present study aims at investigating the application of ultrasound assisted choline chloride (ChCl) – formic acid (FA) deep eutectic solvent (DES) pretreatment of Barley straw. In addition, the efficiency of a wet grinding followed by high intensity ultrasound (HIUS) treatment for production of cellulose nanofibers (CNF) has been evaluated. The DES (using ChCl: FA at 1:9 M ratio) treatment at 45 kHz ultrasound frequency and 3 h of treatment duration resulted in 84.68 ± 1.02 % and 82.96 ± 0.79 % of lignin and hemicellulose solubilisation, respectively. The purification of DES treated solid residue resulted in cellulose with more than 90 % purity. Further, 10 min of wet grinding followed by 40 min of HIUS treatment resulted in more than 80 % nano-fibrillation efficiency. The produced CNF had diameters less than 100 nm in number size distribution and type I cellulose structure. This study confirmed that the developed process offers a sustainable method for producing nanocellulose from agricultural waste.

## Graphical abstract

**Figure:**
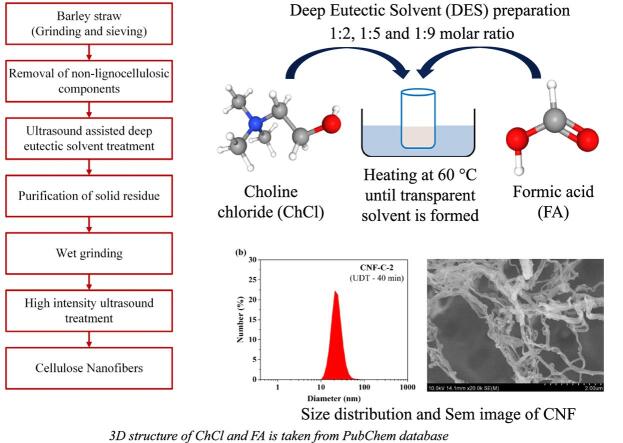

## 1 Introduction

Nanocellulose is derived from cellulosic biomass having diameters in the nanometre range [1], [2]. It has emerged as a significant green material with excellent properties, making it suitable for applications in various fields, including food packaging [3], [4], biomedical and biosensing applications [5], textile industry [6], pharmaceuticals industry [7] and many others [8]. Nanocellulose can be produced from agricultural waste, such as lignocellulosic biomass derived from agro-industrial food waste and crop residues [9], [10], [11]. For instance, Barley straw is a lignocellulose crop residue that can be utilized for production of nanocellulose. According to a study by Fortunati et al. [12], the lignocellulose composition of barley straw comprised of 56.2 % of cellulose, 7 % of hemicellulose and 9.2 % of lignin. The high proportion of cellulose, along with annual regeneration capability of biomass could make barley straw a promising feedstock for production of nanocellulose.

Prior to isolation of cellulose nanomaterials, efficient biomass pretreatment strategies are critical for removing non-cellulosic materials such as lignin and hemicellulose as well as increasing the surface area and solubility of cellulose fibres [13], [14], [15]. In recent years, Deep Eutectic Solvents (DESs), which are prepared by mixing hydrogen-bonding donors (HBD) with hydrogen-bonding acceptors (HBA), have emerged as a promising pathway for pretreatment and conversion of biomass [16]. Usually, DES prepared using an acid HBD tends to exhibit highest fractionation efficiency among other HBD groups. The other HBD groups such as sugar alcohols, alcohols, amines, and amides can be utilised to prepare other categories of DES such as near-neutral DES (e.g., choline chloride – glycerol and choline chloride – glucose) and basic DES (e.g., choline chloride – urea and potassium carbonate – glycerol) [17]. During DES pretreatment of biomass, the lignin fraction can be solubilised in the solvent, and the hemicellulose portion can be broken down into monomers and oligomers under acidic environment [18], [19]. However, cellulose tends to be more resistant and does not dissolve or depolymerize easily in DES. As a result, particularly acidic DES pretreatments can serve as an effective technique for isolating cellulose from biomass [18]. For instance, acidic DES can be prepared by mixing choline chloride (ChCl) as HBA and formic acid (FA) as HBD [20]. In a study, Tian et al. [21] reported that the ChCl – FA (CF) DES treatment of poplar wood resulted in a 76.4 % delignification and an increase in cellulose content from 43.2 % (poplar wood) to 84.7 % (treated sample). In another study, Guo et al. [22] reported that the CF DES treatment of Bamboo waste led to a 95 % delignification which was significantly higher than delignification efficiency (63.2 %) of an alkaline DES (Glycerol – K_2_CO_3_). In another study, Pi et al. [23] reported that using CF DES pretreatment of poplar wood resulted in a 91 % delignification rate and 98 % of cellulose retention. These studies indicated that CF DES is effective in delignification and retains a high amount of cellulose during the pretreatment of various biomass materials. Therefore, CF DES could be further explored as a sustainable solvent for the pretreatment of agricultural waste biomass.

Green emerging technologies for process intensification, such as microwave and ultrasound, could be integrated with DES to enhance the efficiency of biomass fractionation and increase the solubilisation of hemicellulose and lignin fractions [24], [25], [26]. The synergistic effect of ultrasound and DES for pretreatment of different biomass have been reported in few recent studies [26], [27], [28], [29]. However, to the best of our knowledge, no studies have yet explored the ultrasound-assisted DES pretreatment using CF DES for the fractionation of Barley straw biomass. Furthermore, to the best of our understanding, the production of CNF from cellulose fibres derived from Barley straw has not been reported in any studies. Consequently, there is a need to develop sustainable and efficient methods for both the fractionation of Barley straw and the nano-fibrillation of extracted cellulose for CNF production.

On the basis of the research gaps, this study aims to develop a pretreatment method for barley straw biomass using ultrasound-assisted CF DES (prepared using different molar ratios) that could effectively solubilize the lignin and hemicellulose fractions. Furthermore, a mild purification treatment of the DES-treated solid fraction that could result in high-purity cellulose fibres has been proposed. In addition, a chemical-free method for the nano-fibrillation of Barley straw derived purified cellulose fibres (PCF) has been proposed in this study. The nano-fibrillation method, consisting of wet grinding and high-intensity ultrasonication (HIUS) treatment, could effectively reduce the size of PCF to the nanoscale.

## 2 Materials and methods

### 2.1 Materials

The barley straw biomass was purchased from a local shop in Dublin, Ireland. The straw, which was commercially sold by a company, was packaged at the time of procurement and was free of any foreign materials. Therefore, no preprocessing of the biomass, such as washing or drying of Barley straw, was carried out in this study. The chemicals utilized in this work i.e., Sulphuric acid (98 %), Sodium hypochlorite (6–14 %), Choline chloride (≥98 %), Calcium carbonate (≥99.0 %), Formic acid (≥98 %), and Ethanol (≥99.0 %) were procured from Merck Ireland. The HPLC grade sugar standard utilised for biomass composition analysis i.e., D-(+)-Glucose (≥99.5 %), D-(+)-Xylose (≥99.0 %), D-(+)-Mannose (≥99.5 %), D-(+)-Galactose (≥99 %), and L-(+)-Arabinose (≥99.5 %) were purchased from Merck Ireland.

### 2.2 Extraction of non-lignocellulosic components

The grinding (CGOLDENWALL HC-700 grinder, 0.7 kg capacity, 2400 W, 28000 rpm, 3 min grinding time) and sieving operation (Sieve shaker EML 200 premium, VWR, Ireland) of barley straw sample was carried out and particle size of less than 500 μm obtained through this process were utilized for further treatment. The particle size of less than 500 μm of various straw biomass materials have been utilised in different studies [30], [31], [32]. The process of removing ethanol and water-soluble non-lignocellulosic substances from the sample was performed in an ultrasonic bath (Elmasonic xtra ST, Elma Schmidbauer GmbH, Germany) operating at a frequency of 45 kHz and a temperature of 40 °C. Initially, an appropriate quantity of sample was mixed with ethanol in a 1:10 w/v ratio, and this mixture was processed for a duration of 30 min. Following this, the solid residue was recovered via centrifugation (Sorvall ST 16 Centrifuge, Thermo Scientific) at 4000 x g for 10 min. The obtained solid fraction was then mixed with deionized water at a 1:10 w/v ratio and subjected to another 30-minute treatment in an ultrasonic bath operating at a frequency of 45 kHz and a temperature of 40 °C. Following this, the solid fraction was recovered through centrifugation and freeze-dried (Scanvac CoolSafe Freeze Dryer, LaboGene, Denmark) for further treatment. This solid fraction was referred to as extractives-free Barley straw (EFBS) from here on.

### 2.3 Biomass composition analysis

The National Renewable Energy Laboratory (NREL) laboratory analytical procedure for the determination of structural carbohydrates and lignin in biomass was utilised for determining the cellulose, hemicellulose, and lignin content [33]. Required amount of sample was mixed with 72 % Sulphuric acid at a solid-to-acid ratio of 1:10 w/v and the hydrolysis were carried out at 30 °C in a water bath (Sub Aqua Pro, Grant Instruments, United Kingdom) for 1 h. Following this, the acid concentration of the mixture was brought down to 4 % by adding deionised water and the mixture was kept in an autoclave (Ambassador Autoclave, The Rodwell Autoclave Company, United Kingdom) at 121 °C for 1 h. After this, the liquid and solid fraction were separated using vacuum filtration with a filter crucible.

The monosaccharides in the liquid fraction were detected and quantified using an Ultra-High-Performance Liquid Chromatography (UHPLC) instrument equipped with a refractive index detector (RID) (Agilent). A Biorad Aminex HPX-87P column set at 80 °C was utilized for the analysis. The temperature of RID was set at 35 °C. Ultrapure water (0.2 µm filtered and degassed) was utilized as the mobile phase at a 0.6 ml/min flow rate. The injection volume was 10 µL, and the run time for each sample was 20 min. The amount of cellulose in the biomass was quantified as the corresponding amount of glucose, while the hemicellulose quantity was determined as the combined total of xylose, arabinose, galactose, and mannose.

A diluted mixture was prepared by mixing 0.5 ml of liquid fraction with 9.5 ml of water (dilution factor: 20) and the absorbance of the mixture was recorded at 280 nm using a UV–visible spectrophotometer for determining the acid-soluble lignin (ASL) content. The ASL was calculated using a path length of 1 cm, an absorptivity value of 84.8 Lg^−1^cm^−1^ and a dilution factor of 20. On the other hand, the filter crucible containing the solid fraction (acid insoluble residue) was placed in a hot air oven at 105 °C until a constant weight was achieved. Then the ash content of the solid fraction was determined by placing the crucible in a muffle furnace (SNOL, Lithuania) at 575 °C for 24 h for ashing and for calculating the acid-insoluble lignin (AIL) content. The summation of AIL and ASL was reported as the total lignin content. Further, NREL’s laboratory analytical procedure for the determination of ash in biomass was utilised for determining the ash content in the barley straw sample [34].

### 2.4 Biomass pretreatment

#### 2.4.1 DES preparation

The preparation of CF DES was carried out by mixing ChCl with FA at three different molar ratios of 1:2 (CF-A), 1:5 (CF-B) and 1:9 (CF-C). On the basis of the molar ratios, appropriate quantity of each substance was added in a reaction beaker and heated in a water bath set at 60 °C until a transparent solvent was formed. The reaction beaker (a Duran glass bottle) was sealed with a cap to prevent contamination of DES with water while it was placed in the water bath for heating. The preparation of different DES by using water bath as a heating medium has been reported in several studies [35], [36], [37], [38]. The solvent was allowed to cool down and transferred into a storage glass bottle.

#### 2.4.2 Ultrasound-assisted DES treatment (US-DES)

An ultrasonic bath (Elmasonic xtra ST, Elma Schmidbauer GmbH, Germany) was utilized for ultrasound-assisted DES (US-DES) pretreatment of EFBS samples. The ultrasonic bath was designed to be operated at two frequencies (25 kHz and 45 kHz). The independent process factors were ultrasound frequency (25 kHz and 45 kHz), treatment time (3 h and 5 h), treatment temperature (80 °C) and solid-to-DES ratio (1:20 w/w). In various studies, it has been reported that the CF DES showed good efficiency in biomass treatment at high temperature [21], [23]. Therefore, based on the literature review, the maximum controllable temperature (80 °C) in the ultrasonic bath was kept constants for all the experiments. Further, preliminary experiments indicated that a higher solid-to-DES ratio resulted in a highly viscous mixture, which affected the efficient mixing of the biomass with the DES during pretreatment. Here, the higher solid-to-DES ratio meant that the solid content was higher compared to the DES in the solid-DES mixture. Therefore, a lower solid-to-DES ratio (1:20 w/w) was maintained throughout all the experiments. Following the completion of treatment process, the method reported by Muley et al. [39] was used with some modifications to separate and recover the treated solid residue and the DES liquid fraction. The solid residue was recovered by centrifugation at 4000 x g for 10 min. The solid residue underwent a washing process where it was first washed with ethanol three times, then again washed with water three times to remove DES from the treated sample. The ethanol and water used in the washing process were combined with the DES liquid fraction and this liquid mixture was further utilised for recovery of lignin and recycling of DES, ethanol and water. Usually, water has been used as an anti-solvent to precipitate the lignin in the DES liquid mixture [23], [24], [39]. It has been reported that the solubility of lignin decreases as the water content in the DES increases [40]. Therefore, the solid residue was first washed with ethanol to remove the residual DES and prevent the precipitation of lignin during washing steps. Then solid residue obtained after pretreatment was freeze dried for carrying out composition analysis. The hemicellulose solubilisation (%) and lignin solubilisation (%) were calculated using equation (1), (2), respectively to evaluate the effectiveness of the pretreatment process.

(1) $$Hemicellulose\;solubilisation\;\left(\%\right)=\;\left(\frac{\left(H_{I}\times W_{I}\right)-\left(H_{R}\times W_{R}\right)}{H_{I}\times W_{I}}\right)\times 100$$

(2) $$Lignin\;\;solubilisation\;\left(\%\right)=\;\left(\frac{\left(L_{I}\times W_{I}\right)-\left(L_{R}\times W_{R}\right)}{L_{I}\times W_{I}}\right)\times 100$$

Where $W_{I}$ is the initial weight (g) of EFBS sample, $W_{R}$ is the weight (g) of solid residue obtained after US-DES treatment, $H_{I}$ is the hemicellulose content (%) in EFBS sample, $H_{R}$ is the hemicellulose content (%) in solid residue, $L_{I}$ is the lignin content (%) in the EFBS sample, and $L_{R}$ is the lignin content (%) in the solid residue.

To ensure the sustainability of the pretreatment process, the recovery of DES and lignin from the liquid phase (contains ethanol, water and DES) obtained following the US-DES pretreatment was carried out. The recovery of lignin was carried out using the method reported by Pi et al. [23] and Oh et al. [40] with some modifications. Initially, the ethanol present in the liquid fraction was recovered using a rotary evaporator (Rotavapor® R-100, Buchi, Switzerland) at 45 °C and under a low pressure of 150 mbar. Then the remaining liquid fraction (contains DES and water) was mixed with an adequate amount deionised water such that the total amount of water in this mixture was five times that of the initial amount DES used in the pretreatment. This mixture was then left at room temperature for 48 h to allow the precipitation of lignin rich material (LRM). The LRM was recovered by centrifugation at 4000 x g for 10 min and the supernatant was further used for recovery of DES and water. The LRM was then washed twice with distilled water and freeze-dried. The LRM yield with respect to the initial weight of EFBS sample was determined using Equation (3). The lignin estimation method described in section 2.3 was used to determine the purity of lignin (the percentage of lignin in the total solid of LRM). The lignin recovery was determined using the method (Equation (4) reported by Hou et al. [41]. Then the recovery of DES from the liquid fraction (contains DES and water) was performed using a rotary evaporator by removing water. The recovery of FA based DES using rotary evaporator has been reported by Wu et al. [42] and Xie et al. [43]. Initially, the original CF DES (without water) was subjected to evaporation at various temperature and pressure conditions in the rotary evaporator. This was done to identify the optimal parameters for rotary evaporation where there was no mass change of the DES. Subsequently, only water (without DES) was subjected to evaporation under these optimal conditions to verify their effectiveness in removing water. Finally, the optimal parameters (a temperature of 52 °C and a pressure of 65 mbar) were utilized in the rotary evaporator to remove the water from the DES. Both water and DES were collected separately from the rotary evaporator and the DES recovery rate was calculated using Equation (5).

(3) $$LRM\;yield\;\left(\%\right)=\;\left(\frac{Weight\;of\;recovered\;LRM\;(g)}{Initial\;weight\;of\;EFBS\;biomass\;(g)}\right)\times 100$$

(4) $$Lignin\;recovery\;\left(\%\right)=\;\left(\frac{Lignin\;amount\;in\;LRM\;(g)}{Lignin\;amount\;in\;EFBS\;biomass\;(g)}\right)\times 100$$

(5) $$DES\;recovery\;\left(\%\right)=\;\left(\frac{Mass\;of\;DES\;recovered\;(g)\;}{Initial\;mass\;of\;DES\;used\;for\;pretreatment\;(g)}\right)\times 100$$

#### 2.4.3 Purification of DES-treated biomass

The solid residue recovered from the US-DES treatment was purified using a 2 % sodium hypochlorite solution [44]. The process was carried out for 30 min at 60 °C in a water bath using a solid to liquid ratio of 1:10 w/v. After the treatment, the solid fraction was recovered using centrifugation at 4000 x g for 10 min and washed three times with deionised water. The recovered solid fraction through this process was termed as purified cellulose fibres (PCF). The yield of PCF was determined after freeze drying and the purity was analysed using biomass composition analysis protocol as described in section 2.3. The PCF in wet state was further utilised for the preparation of nanocellulose. Using this purification method, the extracted cellulose was transformed into a white pulp as a result of the solubilisation of the remaining hemicelluloses and lignin. The purification process breaks down the phenolic compounds and chromophoric groups found in the lignin molecule, thereby causing the cellulose fibres to become white [44].

### 2.5 Isolation of cellulose nanofibers

The never-dried PCF was utilised for preparation of nanocellulose using a sequential process consisting of wet grinding and high-intensity ultrasonication (HIUS). A quantity of 2 g of PCF (on a dry weight basis) was added to 200 ml of water and wet grinding was performed for 2 min using a kitchen grinder (500 W). The grinding process was repeated for four more times (10 min total) with a three-minute pause implemented between each grinding session to prevent overheating of the grinder. Then the PCF and water were separated using centrifugation at 4000 x g for 10 min and the moisture content of the PCF was measured. Then required quantity of ground PCF was mixed with 500 ml water to maintain a cellulose content of 0.5 g (dry weight basis) in the mixture. This aqueous PCF mixture was nano-fibrillated using HIUS process (2 cm diameter ultrasound probe, 1000 hdT, 20 kHz, 500 W output power, Hislscher, Germany) for 20, 40 and 60 min. The ultrasonic beaker containing the sample was connected to a chiller (set at 1 °C) during HIUS treatment.

The nano-fibrillation efficiency (NFE) was determined using the method reported by Yahya et al. [45], with some modifications. The aqueous PCF mixture treated with HIUS process was centrifuged at 4000 rpm for 10 min to separate the nano-fibrillated material (in the supernatant part) from the non-fibrillated material which settle down (sediment part) [45]. The percentage of sediment part was determined, and this value was subtracted from 100 to calculate the NFE.

### 2.6 Characterization

#### 2.6.1 Dynamic light scattering (DLS)

The particle size, polydispersity index and zeta potential of nanocellulose sample was determined using Dynamic light scattering (DLS) analysis. The nanocellulose sample was diluted ten times and then analysis was performed in a Zetasizer Nano ZS (Malvern Panalytical, US) at a temperature of 25 °C and a scattering angle of 173°. A total of six measurements were performed for each sample.

#### 2.6.2 X-ray diffraction (XRD)

The crystallinity properties of the samples were analysed using the Rigaku Miniflex benchtop X-ray diffractometer, equipped with a monochromatic CuKα radiation source (λ = 1.54059 Å, 40 kV, 15 mA). Analysis was carried out at a 2θ angle range of 5° to 40°, a step width of 0.02°, and a scan speed of 2°/min. The crystallinity index (CI) was calculated (Equation (6) using Segal's method [46].

(6) $$CI\;\left(\%\right)=\frac{I_{002}-{\;I}_{am}}{I_{002}}\times 100$$

Where I_002_ is the maximum intensity (in arbitrary units) of the 002-lattice diffraction at a 2θ angle between 22° and 24° [47]. I_am_ is the minimum diffraction intensity in the same units at a 2θ angle between 18° and 19° [48]. I_002_ represents both amorphous and crystalline regions, whereas I_am_ represents only the amorphous parts [49].

#### 2.6.3 Fourier transform infrared spectroscopy (FTIR)

The functional group and chemical structure of the samples were analysed using the ATR-FTIR Spectrometer (Nicolet iS5, Thermo Scientific, USA). Using the transmittance mode, 64 scans were performed at resolution of 4 cm^−1^ across a range of 4000 – 400 cm^−1^.

#### 2.6.4 Scanning electron microscopy (SEM)

A field emission scanning electron microscope (Hitachi SU-70) was utilised to analyse the morphological characteristics of nanocellulose sample. The analysis was performed at 10 kV of accelerating voltage. The nanocellulose sample was drop cast on an aluminium substrate plate and vacuum drying was carried out. Then the sample was kept on an aluminium stub and coated with a 6 nm layer of platinum using a vacuum sputter coater (Cressington 208HR, UK) prior to SEM imaging.

### 2.7 Statistical analysis

All the experiments were conducted at least in duplicates unless otherwise stated and the results are provided as mean ± standard deviation. Statistical analysis such as One-way ANOVA and Tukey post hoc tests were carried out using SPSS (Version 29, IBM, USA) and the statistically significant difference were considered at p-values < 0.05.

## 3 Results and Discussion

### 3.1 DES pretreatment and cellulose purification

The composition analysis of raw barley straw conducted in this study revealed a cellulose content of 42.50 ± 0.25 %, hemicellulose content of 26.60 ± 0.32 %, lignin content of 18.26 ± 1.01 %, ash content of 2.60 ± 0.05 %, and 10.04 ± 1.64 % of non-lignocellulosic components. The monosaccharides detected in barley straw were glucose, xylose and arabinose. The cellulose content was reported as equivalent of glucose content while the hemicellulose content was detected as the summation of xylose and arabinose. After the removal of non-lignocellulosic components (section 2.2), the cellulose content in the biomass increased to 47.30 ± 0.52 % while the hemicellulose content remained around 26.19 ± 0.04 %. Besides, the lignin content increased to 22.66 ± 0.28 % and the ash content (2.69 ± 0.80 %) was similar to that of raw sample. In addition, the non-lignocellulosic components reduced significantly to 1.17 ± 1.02 %. After the removal of non-lignocellulosic components from raw barley straw, the solid recovered was referred to as extractives free barley straw (EFBS). The EFBS sample was utilised for the pretreatment using US-DES technique.

In general, the use of ultrasound enables a greater penetration of the solvent into the solid cell matrix, which in turn enhances the mass transfer of the targeted compound in the extraction medium [50]. In this study, the US-DES pretreatment of biomass was carried out at 80 °C using different ultrasound frequency (25 kHz and 45 kHz) and treatment time (3 h and 5 h). The composition of solid fraction obtained after US-DES treatment with three different DES such as CF-A DES (ChCl: FA of 1:2 M ratio), CF-B DES (ChCl: FA of 1:5 M ratio), and CF-C DES (ChCl: FA of 1:9 M ratio) are provided in Table 1, Table 2 and Table 3, respectively.

**Table 1:** Composition of solid residue obtained after US-DES treatment at 80 °C using CF-A DES (ChCl:FA of 1:2 M ratio).

Sample code	Ultrasound Frequency (kHz)	Time (h)	Solid Recovery (%)	Cellulose (%)	Hemicellulose (%)	Lignin (%)	Lignin solubilisation (%)	Hemicellulose solubilisation (%)
CF-A-1	25	3	65.46 ± 0.65^b^	66.21 ± 0.88^a^	12.17 ± 0.73^a^	13.71 ± 0.44^c^	60.39 ± 1.67^a^	69.57 ± 2.12^a^
CF-A-2	25	5	64.24 ± 0.74^ab^	71.35 ± 0.87^bc^	11.19 ± 0.70^a^	8.58 ± 0.37^a^	75.69 ± 0.54^c^	72.57 ± 1.40^a^
CF-A-3	45	3	63.34 ± 0.94^ab^	68.38 ± 0.48^ab^	12.60 ± 0.16^a^	11.06 ± 0.31^b^	69.07 ± 1.64^b^	69.52 ± 0.83^a^
CF-A-4	45	5	61.84 ± 0.89^a^	72.77 ± 0.68^c^	11.14 ± 0.45^a^	7.09 ± 0.52^a^	80.63 ± 1.25^d^	73.69 ± 0.68^a^

*Different letters in the same column indicate statistically significant differences (P<0.05).

**Table 2:** Composition of solid residue obtained after US-DES treatment at 80 °C using CF-B DES (ChCl:FA of 1:5 M ratio).

Sample code	Ultrasound Frequency (kHz)	Time (h)	Solid Recovery (%)	Cellulose (%)	Hemicellulose (%)	Lignin (%)	Lignin solubilisation (%)	Hemicellulose solubilisation (%)
CF-B-1	25	3	60.26 ± 0.99^b^	75.21 ± 0.65^a^	9.27 ± 0.50^b^	8.69 ± 0.58^c^	76.89 ± 1.16^a^	78.68 ± 0.79^a^
CF-B-2	25	5	57.28 ± 0.84^ab^	76.36 ± 0.61^a^	8.15 ± 0.84^ab^	5.96 ± 0.66^b^	84.93 ± 1.88^b^	82.18 ± 1.57^ab^
CF-B-3	45	3	55.95 ± 0.96^a^	78.97 ± 0.45^b^	8.04 ± 0.30^ab^	6.63 ± 0.44^bc^	83.65 ± 0.80^b^	82.81 ± 0.93^b^
CF-B-4	45	5	55.33 ± 0.76^a^	79.52 ± 0.71^b^	7.01 ± 0.17^a^	3.58 ± 0.30^a^	91.25 ± 0.85^c^	85.20 ± 0.15^b^

*Different letters in the same column indicate statistically significant differences (P<0.05).

**Table 3:** Composition of solid residue obtained after US-DES treatment at 80 °C using CF-C DES (ChCl:FA of 1:9 M ratio).

Sample code	Ultrasound Frequency (kHz)	Time (h)	Solid Recovery (%)	Cellulose (%)	Hemicellulose (%)	Lignin (%)	Lignin solubilisation (%)	Hemicellulose solubilisation (%)
CF-C-1	25	3	60.41 ± 0.79^b^	74.06 ± 0.56^a^	8.24 ± 0.11^c^	12.91 ± 0.49^c^	65.56 ± 1.75^a^	80.99 ± 0.01^a^
CF-C-2	25	5	58.65 ± 0.90^ab^	76.39 ± 0.50^a^	7.72 ± 0.13^bc^	10.69 ± 0.39^b^	72.32 ± 1.43^b^	82.71 ± 0.02^b^
CF-C-3	45	3	59.29 ± 0.80^ab^	82.50 ± 0.65^b^	7.52 ± 0.25^b^	5.86 ± 0.47^a^	84.68 ± 1.02^c^	82.96 ± 0.79^b^
CF-C-4	45	5	55.54 ± 1.63^a^	83.95 ± 0.62^b^	5.86 ± 0.11^a^	5.50 ± 0.20^a^	86.50 ± 0.89^c^	87.58 ± 0.13^c^

*Different letters in the same column indicate statistically 6significant differences (P<0.05).

The maximum amount of solid recovery (65.46 ± 0.65 %) was obtained after CF-A DES treatment (CF-A-1 sample) at lower frequency (25 kHz) and lowest treatment time (3 h). Keeping the same frequency and treatment time, the solid recovery reduced to nearly 60 % when the acid proportion in the DES was increased (CF-B-1 and CF-C-1 sample). However, when the frequency was maintained at 25 kHz and the treatment time was extended to 5 h, no significant variation in solid recovery was observed among the pairs of samples CF-A-1 and CF-A-2, CF-B-1 and CF-B-2, as well as CF-C-1 and CF-C-2. Further, when the treatment time was held constant at 3 h and the frequency was raised from 25 kHz to 45 kHz, there was a decrease in solid recovery from 60.26 ± 0.99 % (CF-B-1 sample) to 55.95 ± 0.96 % (CF-B-3 sample); however, no substantial differences were noticed between the CF-A-1 and CF-A-3 samples, or between the CF-C-1 and CF-C-3 samples. Furthermore, with the frequency held constant at 45 kHz and the treatment time extended from 3 to 5 h, there were no statistically significant difference observed in solid recovery between the pairs of samples CF-A-3 and CF-A-4, CF-B-3 and CF-B-4, as well as CF-C-3 and CF-C-4. Likewise, when the treatment time was held constant at 5 h and the frequency was raised from 25 to 45 kHz, there were no statistically significant variations noticed in solid recovery between the pairs of samples CF-A-2 and CF-A-4, CF-B-2 and CF-B-4, as well as CF-C-2 and CF-C-4. Nonetheless, the samples CF-B-3, CF-B-4, and CF-C-4 exhibited a minimum solid recovery of approximately 55 %. The solid recovery recorded in the current study was in consistent with the findings from comparable studies reported in literature. In a comparable study, Zhang et al. [51] reported that the around 31.6 % – 54.2 % eucalyptus were dissolved in the CF DES pretreatment process conducted at different temperature and pressure combination. In another study, Yu et al. [52] reported a solid recovery of 76.63 ± 0.12 % from *Triarrhena lutarioriparia* using CF DES treatment conducted at solid–liquid ratio of 1:10 and at 90 ℃ for 3  h. In another study, Oh et al. [40] reported a solid recovery of 46.6 ± 2.3 % following the CF DES pretreatment of pine wood at 130 °C for 6 h.

In the samples treated with DES, the cellulose content exhibited a range, approximately 66 – 73 % for CF-A samples, 75 – 80 % for CF-B samples, and 74 – 84 % for CF-C samples. These results imply that an increase in the proportion of acid in DES corresponds to an increase in cellulose content. When the frequency was held constant at 25 kHz and the treatment duration was extended from 3 to 5 h, there was a significant increase in cellulose content from approximately 66 % (in CF-A-1) to 71 % (in CF-A-2), however, no notable changes were observed between the pairs of samples CF-B-1 and CF-B-2, or between CF-C-1 and CF-C-2. Likewise, keeping the frequency constant at 45 kHz and increasing the time from 3 to 5 h, significant differences in cellulose was observed between CF-A-3 and CF-A-4 samples, however, no substantial differences were seen between CF-B-3 and CF-B-4 samples as well as between CF-C-3 and CF-C-4 samples. On the other hand, when the treatment duration was held constant at either 3 h or 5 h and the frequency was raised from 25 kHz to 45 kHz, there was a significant increase in cellulose content between the pairs of samples CF-B-1 and CF-B-3, CF-B-2 and CF-B-4, CF-C-1 and CF-C-3, as well as CF-C-2 and CF-C-4; however, no substantial difference was noticed between CF-A-1 and CF-A-3 samples as well as between CF-A-2 and CF-A-4 samples. Nonetheless, cellulose content of more than 80 % was observed in samples CF-C-3 and CF-C-4. The increase in cellulose content achieved in the current study was in consistent with the findings from comparable studies reported in literature. For instance, in a comparable study, Zhang et al. [51] reported that the glucan content increased significantly after CF DES pretreatment of eucalyptus (55.0 % − 66.9 % in pretreated solid compared 39.1 % in raw material), indicating the retention of cellulose during the pretreatment. In another study, Oh et al. [40] reported an increase in glucan content from 39.7 ± 0.6 % (untreated pine wood) to 69.3 ± 3.1 % (DES treated pine wood) following the CF DES pretreatment at 130 °C for 6 h.

The solubilization of lignin varied in different DES treated samples, with CF-A samples showing a range of approximately 60 – 81 %, CF-B samples between 76 – 91 %, and CF-C samples from 65 – 86 %. In CF-A, CF-B and CF-C DES treated samples, the lignin solubilisation increased significantly when the treatment time was increased from 3 to 5 h while keeping the frequency constant at 25 kHz. On the other hand, when the frequency was maintained at 45 kHz and the treatment time increased from 3 to 5 h, significant increase in lignin solubilisation was observed in CF-A and CF-B DES treated samples; however, no substantial changes was observed in CF-C DES treated samples. Further, when treatment time was kept constant at 3 h or 5 h and frequency increased from 25 to 45 kHz, the lignin solubilisation increased significantly in CF-A samples, CF-B samples and CF-C samples. All the DES performed well in terms of lignin solubilisation (at least 80 % solubilisation) at higher frequency and higher treatment time; however, maximum lignin solubilisation of around 91.25 % was noticed in CF-B-4 sample (45 kHz and 5 h). In a comparable study, Zhang et al. [51] reported that 74.4 % of lignin was removed in eucalyptus feedstock after CF DES treatment at a high temperature of 120 °C for 90 min. The lignin solubilisation achieved in the current study was higher than the lignin removal from *Triarrhena lutarioriparia* reported by Yu et al. [52], in which they reported that 42.68 ± 0.03 % lignin was removed using CF DES treatment conducted at a low solid–liquid ratio of 1:10 and at 90 ℃ for 3  h. In comparison to the study of Yu et al. [52], the higher lignin solubilization achieved in the current study might be due to the use of a high solid–liquid ratio (1:20) and the use of ultrasound. In another study, Pi et al. [23] reported that when the CF DES pretreatment temperature for poplar wood increased from 60 to 90 °C (long treatment time of 8 h), the delignification efficiency increased from 44 % to 91 %. Similarly, Kohli et al. [24] reported that the lignin extraction from miscanthus feedstocks increased from 4.8 to 82 % on increasing temperature from 60 to 130 °C using CF DES. The increase in temperature decreases the viscosity and enhances the diffusivity of the DES solvent system by disrupting the hydrogen bonds among the solvent components. This facilitates a more efficient interaction between the solvent and the biomass which leads to an increase in the separation of lignin from the biomass [24]. Therefore, in the current study, maximum controllable temperature (80 °C) in the ultrasonic bath was utilised to achieve maximum lignin solubilisation in barley straw.

Among the three primary constituents of lignocellulosic biomass, the hemicellulose fraction is the most vulnerable to degradation when biomass is subjected to chemical and thermal treatment. Therefore, most of the hemicellulose fraction is expected to be removed during DES treatment of biomass at high temperature [53]. The solubilization of hemicellulose varied in different DES treated samples, with CF-A samples showing a range of approximately 69 – 74 %, CF-B samples between 78 – 85 %, and CF-C samples from 80 – 87 %. These findings suggested that the hemicellulose solubilisation increased with the increase in acid proportion in the DES. No significant changes in hemicellulose solubilisation were observed in the CF-A DES-treated samples, despite variations in ultrasonic frequency and treatment time. In CF-B DES treated samples, the minimum hemicellulose solubilisation was observed in CF-B-1 sample (25 kHz and 3 h) whereas maximum solubilisation was recorded in CF-B-4 sample (45 kHz and 5 h). However, no significant difference was observed in hemicellulose solubilisation between CF-B-2, CF-B-3 and CF-B-4 samples. In the samples treated with CF-C DES, there was a significant increase in hemicellulose solubilization when the treatment duration was extended from 3 to 5 h, with the frequency held at either 25 kHz or 45 kHz. Likewise, a significant increase in hemicellulose solubilization was observed when the frequency was raised from 25 to 45 kHz, while the treatment time remained constant at either 3 or 5 h. Nonetheless, a maximum hemicellulose solubilisation of around 87.58 % was achieved in CF-C-4 sample (45 kHz and 5 h). The maximum hemicellulose solubilisation achieved in the current study was comparable to a study by Zhang et al. [51], in which they reported that 95.2 % of xylan was removed in eucalyptus feedstock after CF DES treatment at a high temperature of 120 °C for 90 min. In another comparable study, Pi et al. [23] reported that a hemicellulose removal rate of 76 % in poplar wood was achieved when the CF DES treatment was conducted at 90 °C for a long treatment time of 8 h. The maximum hemicellulose solubilisation achieved in the current study was higher than the hemicellulose removal reported by Yu et al. [52], in which they achieved 46.15 ± 1.06 % of hemicellulose removal from *Triarrhena lutarioriparia* using CF DES treatment conducted at solid–liquid ratio of 1:10 and at 90 ℃ for 3  h. In comparison to the study of Yu et al. [52], the higher hemicellulose solubilization achieved in the current study might be due to the use of a high solid–liquid ratio (1:20) and the use of ultrasound.

The findings of composition analysis of US-DES treated solid residue confirmed that the DES prepared with ChCl and FA at the three different molar ratios performed very well in fractionation of biomass and solubilisation of lignin and hemicellulose. Besides, the DES with a higher molar ratio of HBD (CF-C) demonstrated superior pretreatment efficiency in a shorter time compared to the other two DES prepared with a lower molar proportion of HBD (CF-A and CF-B). In a study, Rani et al. [54] observed a similar trend where maximum dissolution of *Prosopis juliflora* biomass was achieved when the DES (prepared with ChCl and FA) containing higher molar ratio of HBD was used for pretreatment. They also reported that FA, when used as HBD, was effective in simultaneously solubilising the hemicellulose and lignin fractions of the biomass [54]. In another study, Kohli et al. [24] reported that the maximum amount of lignin extraction from miscanthus feedstocks was achieved when FA was used as HBD and ChCl was used as HBA. They observed that FA performed better than other acidic HBD used in the study such as acetic acid, malic acid, and lactic acid [24]. The findings from these studies align with the results observed in the current study.

The mechanism for removal of lignin from biomass is attributed to the cleaving of ether bonds in lignin by DES, which results in the separation of lignin from the lignocellulosic matrix [24], [53], [55]. Besides, lignin tends to dissolve more readily in an environment that is both acidic and highly reactive [54]. The strength of acid in the DES is a crucial parameter and a function of the solvent. The hydrolysis happens in an acidic environment which leads to the disassembly of biomass into individual components such as cellulose, hemicellulose and lignin. This process facilitates the removal of lignin, thereby enhancing the efficiency of acidic DES in biomass fractionation [24]. On the other hand, hemicellulose exhibits a higher reactivity compared to lignin and have the ability to dissolve even in mild and highly viscous solvents with minimal interaction between the feed and the solvent. Furthermore, hemicellulose fraction is prone to hydrolysis in environments that are highly acidic and more reactive [54]. Therefore, a DES that contains a high molar proportion of acid as HBD is likely more appropriate when the concurrent solubilization of lignin and hemicellulose is the objective of the pretreatment process. In addition, the use of acidic DES under ultrasonic assistance in the current study showed excellent efficiency in solubilisation of hemicellulose and lignin. The application of ultrasound might have generated localized pressure in the solvent, leading to rapid molecular movement and enhanced penetration of the solvent into the biomass [56]. This could have subsequently facilitated the efficient disintegration of the lignocellulosic matrix in the barley straw and the release of lignin, hemicellulose, and their degradation products into the solvent, thereby improving the solubilization efficiency of hemicellulose and lignin.

Finally, considering a balance between solid recovery, cellulose content, lignin solubilisation and hemicellulose solubilisation, the CF-C-3 sample (45 kHz and 3 h) was chosen for further downstream processing for nanocellulose production. The purification of CF-C-3 treated samples resulted in a purified cellulose fibres (PCF) yield of 43.39 ± 0.36 % with a purity of 90.62 ± 1.13 %. This PCF sample was referred to as CF-C-PCF and was further utilised for conversion to nanocellulose.

Further, the lignin fraction (i.e., LRM) was recovered from CF-C-3 sample using the method described in section 2.4.2. The LRM yield was found to be 12.58 ± 0.95 % and the purity of lignin in LRM was 84.52 ± 2.59 %. Furthermore, the lignin recovery rate in CF-C-3 sample was 46.85 ± 2.11 %. In a comparable study, Zhang et al. [51] reported a maximum lignin yield of 17.6 g/100 g feedstock with a lignin recovery rate of 60 % in eucalyptus raw materials treated with CF DES at 120 °C for 150 min. Similarly, in another study Oh et al. [40] reported a lignin recovery of around 61 % with a purity of more than 90 % after the treatment of pine wood using CF DES. In another study, Pi et al. [23] reported a 91 % delignification of poplar wood using CF DES treatment and they recovered around 63 % of lignin with a purity of 90 %. Similarly, in the current study, it was observed that the lignin recovery rate was lower than the lignin solubilisation achieved during pretreatment. This indicated that all the lignin that got solubilised during pretreatment process did not completely precipitate and therefore the recovery of unprecipitated lignin was not possible during the lignin recovery process. The incomplete precipitation of lignin may be attributed to the high temperatures causing lignin to degrade into phenolic compounds. These compounds do not precipitate upon the addition of water due to their high-water solubility. Hence, some lignin degradation products may stay dissolved in the DES liquid mixture instead of precipitating with the lignin, thereby reducing the overall lignin recovery rate [24].

The recovery of DES following the completion of pretreatment process was carried out in CF-C-3 sample and the recovery rate of CF-C DES was found to be around 85.67 ± 3.60 %. The recovery rate of formic acid-based DES after the treatment process have been reported in various studies. For instance, Wu et al. [42] reported that the recovery rate of CF DES was over 95 % after the first cycle of treatment process. Similarly, in another study, Oh et al. [40] reported that the recovery efficiency of a DES (prepared with ChCl:Lactic acid:FA) was over 90 % after five cycle of treatment of pine wood. In addition to the DES recovery, the ethanol and water used in the pretreatment process were recovered and could be reused, thereby making the pretreatment process sustainable.

### 3.2 Nano-fibrillation efficiency and Dynamic light scattering (DLS) analysis

**Fig. 1:**
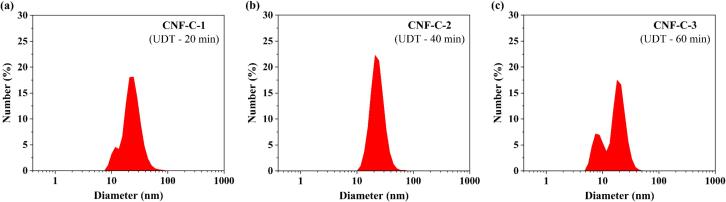
Number size distribution of CNF-C samples (Plotted using average values of 6 measurements).

The nano-fibrillation efficiency (NFE) and DLS characterisation of CNF-C samples produced from CF-C-PCF sample at difference ultrasound defibrillation time (UDT) are provided in Table 4. In addition, the number size distribution plot of CNF-C-1 (20 min UDT), CNF-C-2 (40 min UDT) and CNF-C-3 (60 min UDT) samples are depicted in Fig. 1. The size of the CNF diameters ranged from 9 – 80 nm, 12 – 68 nm and 6 – 44 in CNF-C-1, CNF-C-2 and CNF-C-3 samples, respectively. These results indicated the maximum diameter size reduced with the increase in UDT. Besides, the peak diameter also reduced from 25 nm to 18 nm with the increase in UDT from 20 to 60 min. When CNF was produced using 40 min of UDT, the peak diameter was observed to be 21 nm, with approximately 22.2 ± 4.6 % of nanoparticles contributing to this peak diameter in number size distribution. Nonetheless, the number size distribution confirmed that the all the CNF samples exhibited diameters less than 100 nm.

**Table 4:** Nano-fibrillation efficiency and DLS characterisation CNF-C produced from CF-C-PCF sample (Number size distribution, Polydispersity index and Zeta potential).

**Sample / DLS parameters**		**CNF-C-1** **(UDT – 20 min)**	**CNF-C-2** **(UDT – 40 min)**	**CNF-C-3** **(UDT – 60 min)**
Nano-fibrillation efficiency (%)		61.34 ± 3.41^a^	82.64 ± 2.98^b^	84.13 ± 3.36^b^
*Size Range (nm)		9 – 80	12 – 68	6 – 44
*Peak Diameter (nm)		25	21	18
*Contribution of peak diameter (%)		18.1 ± 8.7	22.2 ± 4.6	17.4 ± 12.4
Polydispersity index (PDI)		0.426 ± 0.1^a^	0.405 ± 0.070^a^	0.373 ± 0.039^a^
Zeta potential (mV)		−40.23 ± 4.15^a^	−46.17 ± 3.92^a^	−39.37 ± 3.91^a^

*Obtained from number size distribution of DLS particle size analysis; UDT – Ultrasound defibrillation time. Different letters in the same row indicate statistically significant differences (P<0.05).

On the other hand, there was a significant improvement in NFE with the increase in UDT. The NFE increased from around 61.34 % (CNF-C-1) to 82.64 % (CNF-C-2) with the increase in UDT from 20 to 40 min. However, further increasing the UDT from 40 to 60 min did not substantially increase the NFE. At higher UDT of 60 min (CNF-C-3 sample), the appearance of the nano-fibrillated sample was greyish in colour which might be attributed to the carbonisation of cellulose as a result of high intensity ultrasound treatment for a long period. Further, the process of converting PCF to CNF can be attributed to the phenomenon of acoustic cavitation. This occurs when the application of ultrasound leads to the creation, growth, and eventual implosion of microbubbles in an aqueous solution. This implosion results in a violent collapse that generates microjets and shock waves on the surfaces of the PCF. This action erodes the fiber surfaces, causing them to split in the axial direction. The impact of sonification can disintegrate the relatively weak interfaces between the nanofibers, which are primarily bonded to each other by hydrogen bonds. As a result, the ultrasonic treatment can progressively break down the micron-sized cellulose fibres into nanofibers [57].

The polydispersity index (PDI) value (between 0 and 1) of a colloidal suspension is a parameter that indicates the degree of homogeneity of the suspension. A lower value of PDI signifies a more homogeneous distribution of particle sizes in the system [58]. The PDI value of CNF samples did not change significantly with the increase in UDT from 20 to 60 min. Maximum PDI of around 0.426 was recorded in CNF-C-1 sample while minimum PDI of around 0.373 was recorded in in CNF-C-3 sample. Nonetheless, the PDI of all three CNF samples were under 0.5, indicating that the nanomaterials had a relatively uniform and narrower size distribution. Besides, the zeta potential (ZP) of all the three CNF samples were below −30 mV. There was no significant difference observed in ZP between CNF-C-1, CNF-C-2, and CNF-C-3 samples. A minimum ZP (in terms of average value) of around −46.17 mV was measured for CNF-C-2 sample. It was clear that all the CNF samples had a negative charge. The higher negative charges of CNF samples are indicative of the nanofibers possessing strong electrical stability [59]. During the process of ultrasound treatment, the collapsing of fine bubbles leads to the formation of radicals (${OH}^{.},\;O_{2}^{-.}$) and promoting oxidation [60], [61]. The ultrasound treatment facilitated improved agitation of the suspension, resulting in increased interaction between oxygen radicals and CNF. This interaction likely favoured the development of a negative charge on the surface of the CNF, which can be attributed to the partial oxidation of the nano particles [61], [62], [63].

Considering a balance between the NFE, size distribution parameters, PDI and ZP values, the CNF-C-2 sample was chosen as the best condition to produce CNF from CF-C-PCF sample. Therefore, CNF-C-2 sample was further characterized using SEM, FTIR and XRD techniques.

### 3.3 Scanning electron microscopy (SEM)

**Fig. 2:**
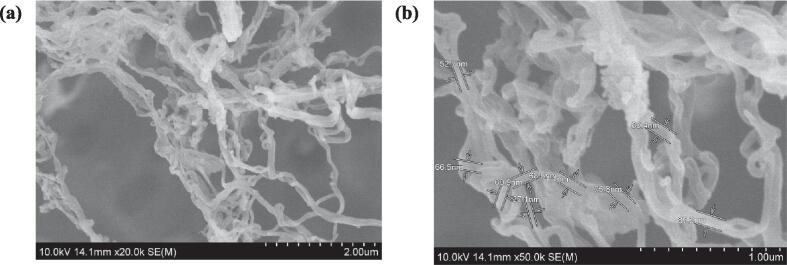
SEM images of CNF-C-2 sample at a magnification of 20 k and 50 k, respectively.

The morphology of the CNF is a crucial factor that needs to be controlled during the synthesis process. The shape and structure of the CNF are heavily influenced by the source of the cellulose and the methods used in its synthesis [64]. The morphology of the CNF-C-2 sample was analysed through SEM at a magnification of 20 k (Fig. 2.a) and 50 k (Fig. 2.b). From Fig. 2.a, it was observed that the CNF sample exhibited a web-like network structure which was comprised of cellulose nano fibrils that were randomly intertwined. Similar morphologies of CNF have been documented in multiple studies where the cellulose nanomaterials were derived from different types of agricultural straw waste, including rice straw [65], wheat straw [66], and rapeseed straw [48].

In Fig. 2.b, it was observed that there was presence of some larger fibril bundles or aggregates in the CNF sample. In CNF samples that have not undergone any chemical modifications, the occurrence of a random number of larger fibres or aggregates is commonly observed [67]. In addition, the process of preparing samples for SEM analysis could lead to particle aggregation. For instance, some drying techniques necessary to prepare wet samples for SEM analysis can cause CNF to form aggregates to a certain extent [64]. However, for numerous applications, it is preferable to prevent the aggregation of nanofibers [68]. Nonetheless, the application of the wet grinding technique followed by a 40 min HIUS treatment resulted in the isolation of CNFs with widths primarily under 100 nm, as shown in Fig. 2.b.

### 3.4 Fourier transform infrared spectroscopy (FTIR)

**Fig. 3:**
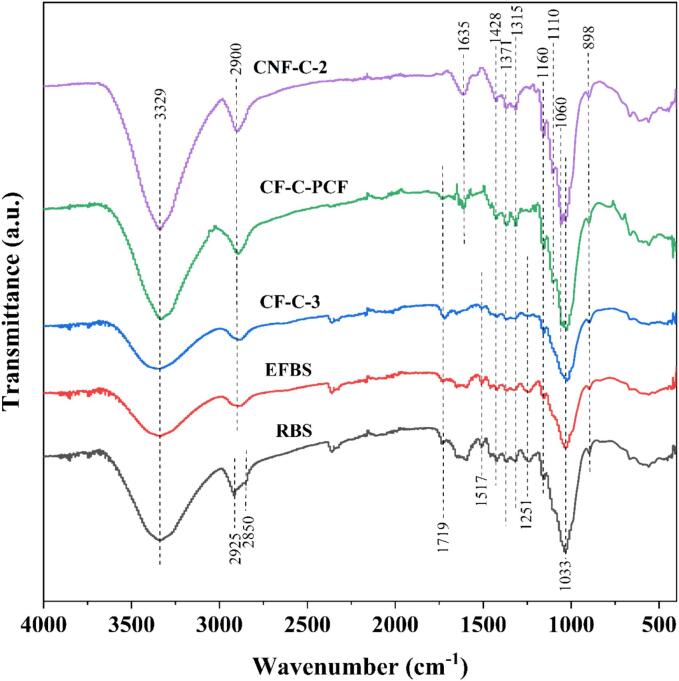
FTIR spectra of RBS, EFBS, CF-C-3, CF-C-PCF and CNF-C-2 samples.

The chemical properties of the barley straw subjected to each unit operation for production of nanocellulose were investigated using FTIR spectroscopy. The FTIR spectra for the RBS, EFBS, CF-C-3, CF-C-PCF and CNF-C-3 samples are illustrated in Fig. 3. In all the samples, the a broad band observed at around 3329 cm^−1^ is indicative of the stretching vibrations in hydroxyl (OH) groups [69]. A strong band at 2925 cm^−1^ was detected in RBS sample, which was shifted slightly shifted to around 2900 cm^−1^ (detected in rest of the samples) after the treatment of biomass. The band detected at around 2900 cm^−1^ is associated with the C–H symmetrical and asymmetrical stretching vibrations in cellulose structures [70]. Further, the vibration at 2850  cm^−1^ originating from C–H stretching in lignin and waxes was only observed in RBS sample and was disappeared after the RBS sample was subjected to ethanol and water-soluble extractives removal process [71].

Characteristics cellulose peaks were observed in FTIR spectra of all samples. The observed peak at 1110 cm^−1^ (detected in CF-C-PCF and CNF-C-2 samples) is assigned to the C–O stretching vibration and the peak at 1160 cm^−1^ is linked to the C–O–C stretching vibration of the pyranose ring present in polysaccharides [72], [73]. The peak observed at 1371 cm^−1^ corresponds to the asymmetric deformation of the C–H bond and symmetric stretching of the C–O bond within the polysaccharide aromatic rings of cellulose. Furthermore, the peak detected at 1428 cm^−1^ is assigned to the CH_2_ symmetric bending of cellulose while the peak at 1315 cm^−1^ represents the C–H bending of cellulose [74]. The peak observed at 1060  cm^−1^ (detected in CF-C-PCF and CNF-C-2 samples) and 898  cm^−1^ are attributed C–O stretching, and C–H rocking vibrations, respectively [75]. The sharp band observed at 898 cm^−1^ in all the samples is indicative of the β-glycosidic bonds that connect the monosaccharides of cellulose. This implies that the glucose units, which constitute the cellulose backbone, are interconnected through β-form bonds [76]. The strong band at 1033 cm^−1^ is assigned to the C–O stretching modes of the hydroxyl and ether groups found in cellulose [72]. It can be noted that the produced nanocellulose (CNF-C-2 sample) showed peaks at 898 cm^−1^, 1110 cm^−1^, 1160 cm^−1^, and 1428 cm^−1^ which confirmed that the CNF retained primarily the structure of cellulose I [74].

The peak detected around 1635  cm^−1^ can be assigned to the bending mode stretch of the absorbed water. This peak was not detected in RBS, EFBS and CNF-C-3 samples; however, it was observed in CF-C-PCF and CNF-C-2 samples which indicated that the purified cellulose and nanocellulose have strong affinity for water [76]. It has been reported that Cellulose has a tendency to be hydrophilic, leading to a strong molecular interaction with water [77].

Moreover, in the RBS and EFBS samples, the band observed at 1517 cm^−1^ is assigned to the C=C stretching of aromatic ring of the lignin whereas a sharp peak observed at 1251 cm^−1^ is attributed to the C=O stretching bands of hemicellulose and lignin. However, the intensity of these peak reduced in the CNF-C-3 sample and was entirely absent in the CF-C-PCF and CNF-C-2 samples [78]. In addition, the band observed at around 1719  cm^−1^ is associated with the C=O stretching of acetyl and uronic ester groups found in hemicellulose, or the ester linkage of carboxylic groups of ferulic and p-coumaric acids present in lignin [79]. Following the purification treatment of the US-DES treated solid residue, the intensity of this band diminished. Furthermore, in the CNF-C-2 sample, this band was found to be disappeared. This suggests that the hemicellulose and lignin fractions were dissolved during the successive unit operations subjected to barley straw for nanocellulose production.

### 3.5 X-ray diffraction (XRD)

**Fig. 4:**
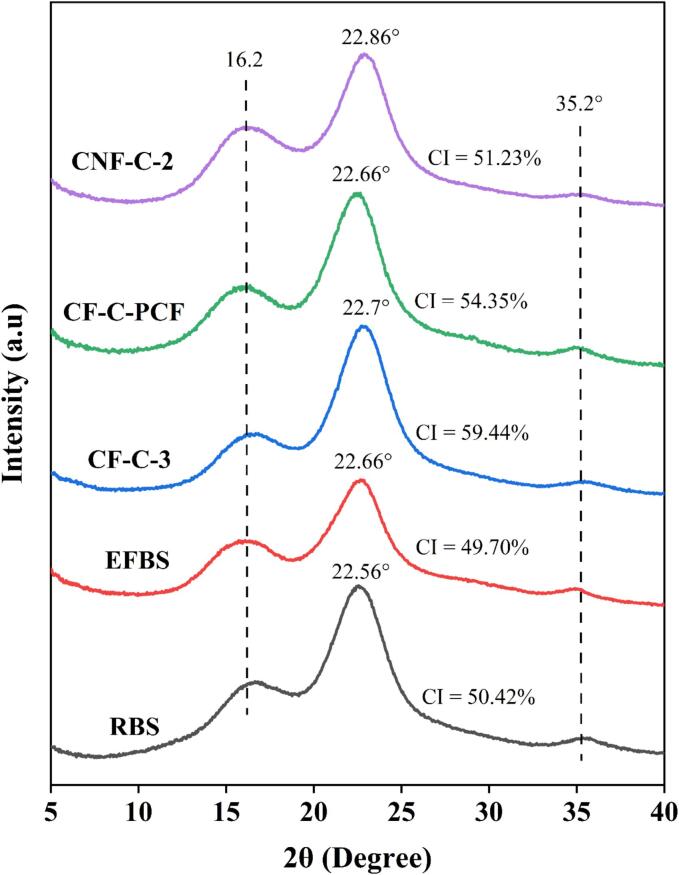
XRD patterns of RBS, EFBS, CF-C-3, CF-C-PCF and CNF-C-2 samples.

X-ray diffraction (XRD) analysis was conducted to find out the crystalline properties of the RBS, EFBS, CF-C-3, CF-C-PCF and CNF-C-2 and the XRD patterns of these samples are illustrated in Fig. 4. In all the samples, a strong and sharp peak were obtained between 2θ angle of 22° to 24° which is associated with the amorphous and crystalline fractions of cellulose. These peaks are linked to the (002) crystallographic plane and are typically seen in the structure of cellulose type I [47]. Within the (002) plane, the RBS sample showed maximum intensity at 22.56°. After the removal of non-lignocellulosic components from biomass, this peak shifted slightly to 22.66° (EFBS sample). Further treatment of the EFBS sample with US-DES method led to a slight peak shift to 22.7°. Further, after purification process, the CF-C-PCF sample exhibited its highest diffraction intensity at 22.66°, while the CNF-C-2 sample reached its peak intensity at 22.86°. Similarly, in all the samples, a clear and intense peak near to 2θ values of 16.2° was observed, which represents the (110) crystallographic plane and is associated with the type I cellulose structure [80]. Furthermore, weak diffraction peaks at 2θ values near to 35.2° were observed in all the samples. The peaks obtained at these diffraction angles are linked to the (004) plane and are indicative of the structure of cellulose I [69], [81]. These results verified that the structure of type I cellulose was preserved throughout the CNF production process from barley straw. The type I cellulose is the native form of cellulose predominantly found in most plant cell walls and structures [82].

The crystallinity index (CI) is an crucial factor to understand the relative amount of crystalline material in cellulose fibres and its derivatives such as CNF [83]. The CI of RBS (50.42 %) and EFBS (49.70 %) samples were almost similar; however, after the US-DES pretreatment, the CI increased significantly to 59.44 % (CF-C-3 sample). The substantial solubilisation of non-cellulosic substances such as hemicellulose and lignin during US-DES treatment might have positively influenced the increase in CI. Furthermore, the CI of CF-C-PCF and CNF-C-2 samples were estimated to be around 54.35 % and 51.23 %, respectively. The CI of CNF isolated from Barley straw is similar to that of CI of CNF isolated from different agricultural waste such as rice straw (CI: 50.57 %) [84], Soybean straw (CI: 50 %) [85], and Durum Wheat straw (CI: 52 %) [86]. Further, it was observed that the CI reduced after the purification treatment and the subsequent downstream treatment for production of CNF using HIUS process. Similar reduction in CI of cellulose nanoparticles was reported in a study where the CI reduced substantially from 72 % (no ultrasound) to 58 % (600 W for 60 min) after ultrasonication [62]. The reduction in CI of nanocellulose might be attributed to the cleavage of the glycosidic bonds in cellulosic fibres and subsequent collapse of the crystalline region as a result of the intense cavitation forces generated by ultrasound treatment [61].

## 4 Conclusion

In conclusion, the application of ultrasound-assisted choline chloride-formic acid deep eutectic solvent treatment, followed by wet grinding and high-intensity ultrasound treatment, proved to be highly effective in the delignification, hemicellulose solubilisation and nano-fibrillation processes. More than 80 % of lignin and hemicellulose were solubilised using the DES (using ChCl: FA at 1:9 M ratio) treatment at 45 kHz ultrasound frequency and 3 h of treatment time. Further, more than 80 % nano-fibrillation efficiency was achieved using 10 min of wet grinding followed by 40 min of high intensity ultrasonication. The produced CNF had good stability in aqueous suspension and had retained the structure of native cellulose found in plant materials. These findings successfully demonstrated the efficiency of the developed method to produce cellulose nanofibers from agricultural waste.

## CRediT authorship contribution statement

**Dileswar Pradhan:** Writing – review & editing, Writing – original draft, Methodology, Investigation, Data curation, Conceptualization. **Swarna Jaiswal:** Writing – review & editing, Validation, Supervision. **Brijesh K. Tiwari:** Writing – review & editing, Resources. **Amit K. Jaiswal:** Writing – review & editing, Validation, Supervision, Funding acquisition, Conceptualization.

## Declaration of competing interest

The authors declare that they have no known competing financial interests or personal relationships that could have appeared to influence the work reported in this paper.
